# Promoting healthy lifestyle behaviours in child and adolescent mental healthcare: reflections from a collaborative nursing and psychology initiative

**DOI:** 10.1177/17449871261442365

**Published:** 2026-05-06

**Authors:** Eva H Larsson, Albert Westergren

**Affiliations:** Licensed Psychologist and Specialist in Neuropsychology, Child and Adolescent Psychiatry, Skåne Regional Council, Sweden; Professor, Faculty of Health Sciences, Kristianstad University, Sweden; Health Care Strategist, Office for Psychiatry, Habilitation and Technical Aids, Skåne Regional Council, Sweden

## Introduction

Healthy lifestyle behaviours, including sufficient sleep, balanced nutrition, physical activity, meaningful leisure activities and balanced screen use are widely recognised as fundamental to mental health across the life course. In child and adolescent mental healthcare, these behaviours are often described as prerequisites for recovery, resilience and longer-term well-being (Firth et al., 2020). Yet despite broad agreement about their importance, lifestyle behaviours frequently occupy an ambiguous position in everyday clinical practice: acknowledged in principle, but often overshadowed by symptom-focused assessment and time-limited treatments.

Child and Adolescent Psychiatry (CAP) services face rising demand, constrained resources and increasingly complex presentations, at least within the Swedish context (Sveriges Kommuner och Regioner, 2025). In this reality, there is a pressing need for interventions that are clinically useful, family-centred, scalable and feasible to deliver early in care pathways. Low-threshold, structured health-promotion formats that fit within routine care may help bridge the gap between evidence and everyday practice.

We reflect here on a structured workshop developed and evaluated within CAP in Region Skåne, Sweden, designed to engage young people with mental health problems and their close relatives in actionable lifestyle changes. Drawing on a regional development and evaluation report (Larsson, 2026), we reflect on how such initiatives can be operationalised in clinical practice and what they imply for nursing roles and interprofessional collaboration.

## Lifestyle behaviours as a clinical foundation in child and adolescent mental healthcare

A substantial body of research links lifestyle behaviours to mental health outcomes in children and adolescents. Regular physical activity is associated with reduced depressive symptoms and improved well-being (Rodriguez-Ayllon et al., 2019), whereas insufficient or irregular sleep is linked to emotional dysregulation, anxiety and depression (Paruthi et al., 2016; Short et al., 2019). Healthier dietary patterns have been associated with better psychological well-being and a reduced risk of depressive symptoms among adolescents, whereas higher consumption of foods high in sugar and fat has been linked to poorer mental health outcomes (Glabska et al., 2020; Ljungberg et al., 2020; Xu et al., 2020). Problematic or excessive screen use has been associated with poorer sleep, attention difficulties and increased internalising symptoms, particularly among vulnerable groups (Nutley and Thorell, 2021).

Rather than treating lifestyle factors as background variables, clinicians should regard them as modifiable determinants that interact with symptoms, family dynamics and treatment engagement. From a nursing perspective, this is not an abstract claim: nurses routinely work with routines, environments and daily rhythms, supporting families in translating health knowledge into sustainable practices, as articulated in national guidance for preventive care (Socialstyrelsen, 2024). This practical orientation positions nursing as a core contributor to translating lifestyle evidence into everyday mental healthcare.

## The workshop: design, aims and core components

The workshop was developed as a low-threshold, step-one intervention intended for adolescents (approximately 11 years and older) and their close relatives, excluding cases with eating disorders or severe eating-related problems. The format combines brief psychoeducation, structured reflection and a practical planning exercise designed to result in one concrete, family-specified behaviour goal (Larsson, 2026).

The workshop focused on five lifestyle domains, all of which are supported by evidence linking them to mental health:

• Sleep, which plays a critical role in emotional regulation, cognitive functioning and mental well-being (Paruthi et al., 2016; Short et al., 2019).
• Physical activity, associated with reduced depressive symptoms and improved quality of life in children and adolescents (Pascoe et al., 2020; Rodriguez-Ayllon et al., 2019).
• Diet and nutrition, where healthier dietary patterns are linked to better mental health outcomes (Glabska et al., 2020; Ljungberg et al., 2020).
• Screen use, where excessive or dysregulated use has been associated with sleep disturbances and poorer mental health, whereas moderated and supported use may have neutral or positive effects (Nutley and Thorell, 2021).
• Meaningful and social leisure activities, including shared enjoyable activities, which support emotional regulation, social connectedness and coping (Berger et al., 2018; Staempfli, 2007).

These domains are interconnected and mutually reinforcing. For example, excessive screen use may disrupt sleep, poor sleep may reduce capacity for physical activity and low levels of social engagement may increase sedentary behaviour. The workshop explicitly addressed these interconnections and encouraged families to focus on one manageable change within the broader lifestyle system, with the potential to influence other lifestyle domains (Larsson, 2026).

## What the evaluation found: a practice snapshot

The evaluation showed that adolescents and relatives perceived the workshop as meaningful, relevant and useful. Participants reported increased understanding of the relationship between lifestyle behaviours and mental health and described gaining practical strategies for change. Most families identified at least one lifestyle domain to work on, and follow-up one month later suggested that many had initiated changes to varying degrees (Larsson, 2026).

Clinicians highlighted feasibility challenges, including recruitment difficulties, time constraints and the need for clearer structure and age adaptation. These findings reflect common implementation challenges when introducing new interventions in high-demand services (Socialstyrelsen, 2012).

## Family-centred practice: why the family orientation matters

Lifestyle behaviours in childhood and adolescence are embedded in family routines, parental capacity and social context. By inviting both adolescents and their relatives, the workshop adopts a family-centred approach that aligns with principles of person-centred care and shared decision-making as articulated in national healthcare guidance (Socialstyrelsen, 2024).

Differences between adolescents’ and relatives’ perspectives were evident: adolescents were often satisfied with the workshop as delivered, whereas relatives more frequently requested additional structure and guidance. These findings underline the importance of flexibility and sensitivity to different needs within the same intervention (Larsson, 2026).

## Interprofessional collaboration: complementary roles of psychology and nursing

The workshop was developed and delivered through collaboration between psychologists, nurses and other professionals. Psychological expertise shaped the psychoeducational content and behaviour-change principles, whereas nursing contributions were evident in the focus on routines, practical planning and follow-up.

This collaboration illustrates how psychology and nursing can complement each other in lifestyle-focused interventions. Nurses’ strengths in continuity, coordination and everyday problem-solving are particularly relevant for sustaining change beyond initial intervention sessions.

## Feasibility, sustainability and the nursing contribution

Feasibility challenges identified in the evaluation, including recruitment, time constraints and integration into routine workflows, are well known in nursing practice within child and adolescent mental health services (Socialstyrelsen, 2012).

The workshop does not aim to transform all lifestyle behaviours simultaneously. Instead, it offers a structured starting point that aligns with nursing approaches to incremental behaviour change. Nurses are often well positioned to support sustainability through follow-up contacts, coordination with other services and ongoing adjustment of goals.

## Implications for nursing practice and service development

This practice-based example highlights several implications for nursing:

• Lifestyle behaviours should be recognised as legitimate clinical targets in CAP, not merely as background advice.
• Low-threshold, family-oriented formats can support health promotion even in resource-constrained settings.
• Nursing roles in follow-up, coordination and adaptation should be explicitly articulated when implementing such interventions.
• Sustainability is likely to be enhanced when interventions are embedded in ongoing nursing contacts rather than delivered as one-off events.

## Concluding reflection

Lifestyle behaviours are often described as ‘basic’ in child and adolescent mental healthcare, yet they are embedded in complex developmental, relational and structural contexts. The workshop described here illustrates how structured, low-threshold lifestyle interventions can be integrated into routine care through interprofessional collaboration and teamwork. By drawing on nursing strengths in continuity, practicality and family-centred care, such initiatives can contribute meaningfully to early, preventive mental health work.

**Key takeaways for nursing practice**

• Encourage families to focus on one realistic, concrete lifestyle change at a time.
• Use nursing continuity to support follow-up and adaptation over time.
• Ensure adolescents’ perspectives are prioritised in family-based interventions.
• Use structured, standardised materials to enhance feasibility in busy clinical settings.
• Leverage interprofessional collaboration, particularly between nursing and psychology, to integrate lifestyle-focused interventions into routine care.
